# Genetic Study in Korean Pediatric Patients with Steroid-Resistant Nephrotic Syndrome or Focal Segmental Glomerulosclerosis

**DOI:** 10.3390/jcm9062013

**Published:** 2020-06-26

**Authors:** Eujin Park, Chung Lee, Nayoung K. D. Kim, Yo Han Ahn, Young Seo Park, Joo Hoon Lee, Seong Heon Kim, Min Hyun Cho, Heeyeon Cho, Kee Hwan Yoo, Jae Il Shin, Hee Gyung Kang, Il-Soo Ha, Woong-Yang Park, Hae Il Cheong

**Affiliations:** 1 Department of Pediatrics, Seoul National University College of Medicine, Seoul 03080, Korea; eujinpark@hallym.or.kr (E.P.); medicalpooh@hanmail.net (Y.H.A.); kanghg@snu.ac.kr (H.G.K.); ilsooha@snu.ac.kr (I.-S.H.); 2 Department of Pediatrics, Kangnam Sacred Heart Hospital, Hallym University College of Medicine, Seoul 07441, Korea; 3 Samsung Genome Institute, Samsung Medical Center, Seoul 06351, Korea; spinelyc@gmail.com (C.L.); bionkdk@gmail.com (N.K.D.K.); woongyang.park@samsung.com (W.-Y.P.); 4 GENINUS Inc., Seoul 05836, Korea; 5 Department of Pediatrics, Asan Medical Center Children’s Hospital, University of Ulsan College of Medicine, Seoul 05505, Korea; yspark@amc.seoul.kr (Y.S.P.); pedkid@gmail.com (J.H.L.); 6 Department of Pediatrics, Pusan National University Children’s Hospital, Yangsan 50612, Korea; pedksh@gmail.com; 7 Department of Pediatrics, Kyungpook National University School of Medicine, Daegu 41944, Korea; chomh@knu.ac.kr; 8 Department of Pediatrics, Samsung Medical Center, Sungkyunkwan University School of Medicine, Seoul 06351, Korea; heeyeon1.cho@samsung.com; 9 Department of Pediatrics, Korea University Guro Hospital, Seoul 02841, Korea; guroped@korea.ac.kr; 10 Department of Pediatrics, Yonsei University College of Medicine, Seoul 03722, Korea; shinji@yuhs.ac; 11 Division of Pediatric Nephrology, Severance Children’s Hospital, Seoul 03722, Korea; 12 Department of Molecular Cell Biology, Sungkyunkwan University School of Medicine, Suwon 16419, Korea

**Keywords:** steroid-resistant nephrotic syndrome, focal segmental glomerulosclerosis, genetic analysis

## Abstract

Steroid-resistant nephrotic syndrome (SRNS) is one of the major causes of end-stage renal disease (ESRD) in childhood and is mostly associated with focal segmental glomerulosclerosis (FSGS). More than 50 monogenic causes of SRNS or FSGS have been identified. Recently, the mutation detection rate in pediatric patients with SRNS has been reported to be approximately 30%. In this study, genotype-phenotype correlations in a cohort of 291 Korean pediatric patients with SRNS/FSGS were analyzed. The overall mutation detection rate was 43.6% (127 of 291 patients). *WT1* was the most common causative gene (23.6%), followed by *COQ6* (8.7%), *NPHS1* (8.7%), *NUP107* (7.1%), and *COQ8B* (6.3%). Mutations in *COQ6*, *NUP107*, and *COQ8B* were more frequently detected, and mutations in *NPHS2* were less commonly detected in this cohort than in study cohorts from Western countries. The mutation detection rate was higher in patients with congenital onset, those who presented with proteinuria or chronic kidney disease/ESRD, and those who did not receive steroid treatment. Genetic diagnosis in patients with SRNS provides not only definitive diagnosis but also valuable information for decisions on treatment policy and prediction of prognosis. Therefore, further genotype-phenotype correlation studies are required.

## 1. Introduction

Childhood onset nephrotic syndrome (NS) generally responds well to conventional oral corticosteroid therapy, and steroid-sensitive NS has a benign long-term renal prognosis even though it shows frequent relapses. However, 10–20% of pediatric NS cases are steroid-resistant nephrotic syndrome (SRNS), which does not achieve remission by oral corticosteroid therapy. SRNS is one of the most common causes of end-stage renal disease (ESRD) in childhood, and the common pathological finding in SRNS is focal segmental glomerulosclerosis (FSGS) [1].

Furthermore, SRNS is highly heterogeneous both phenotypically and genetically and can occur at any age, manifesting as isolated proteinuria or fully developed NS. In addition, various kinds of syndromic diseases may manifest SRNS as a renal phenotype along with characteristic extrarenal phenotypes. To date, at least 50 monogenic causes of SRNS or FSGS have been identified, and novel causative genes are constantly being discovered [2].

Recent advances in next-generation sequencing (NGS) techniques make genetic diagnosis more feasible in SRNS patients. Recent large cohort studies [3,4,5,6] revealed genetic mutations in approximately 30% of pediatric patients with SRNS/FSGS. Benefits of genetic diagnosis in patients with SRNS include providing definitive diagnosis and valuable information for decisions on treatment policy and prediction of prognosis.

Genetic testing modalities currently used for SRNS include Sanger sequencing, targeted exome sequencing (TES) with gene panels, whole exome sequencing (WES) and whole-genome sequencing (WGS) [7,8,9,10,11]. Sanger sequencing can be effectively applied to test a single or few candidate genes using a gene-by-gene approach. Candidate genes can be easily identified clinically in patients with typical extrarenal phenotypes of syndromic diseases. In addition, one of the four candidate genes (*NPHS1, NPHS2, WT1*, and *LAMB2*) can be identified in more than 85% of patients with congenital onset [12,13]. Sanger sequencing is still regarded as the gold standard, and variants detected by NGS should be confirmed by Sanger sequencing [14]. In patients whose candidate genes are not identified clinically, gene-by-gene Sanger sequencing of more than 50 known candidate genes for SRNS is a time-consuming and costly test. Instead, TES with gene panels can provide a rapid and cost-effective test by sequencing a set of all known genes relevant to SRNS simultaneously. Notably, some patients with syndromic disorders manifest no or mild/atypical extrarenal phenotypes. In these cases, the suspected underlying genetic syndrome cannot be identified clinically, but TES is able to detect the disease-causing mutations [14]. However, TES cannot detect mutations in novel or phenocopying genes unless these genes are included in the panels. Another disadvantage of TES is rapidly outdated gene panels due to the continuous discovery of novel disease-causing genes. Despite these drawbacks, TES with gene panels is currently considered the most cost-effective approach for indication-driven mutation analysis [10,14].

Compared with TES gene panels, WES is an unbiased approach to genetic diagnosis by sequencing entire exons, i.e., all protein-coding regions of the genome. Therefore, WES can identify not only known gene mutations but also novel disease-causing genes. Current limitations of WES include issues with lower coverage of some exons, high costs and difficulty in handling enormous amounts of data [8,9,10,11,14]. A more complete per-base coverage can be provided by WGS, but the cost and interpretation issues are larger than in WES [8,9,14]. Currently, Sanger sequencing or TES is used as the first-line test for the genetic diagnosis of SRNS, and WES or WGS can be used when the first-line test shows negative results [8,9,10,11,14]. In this study, 127 patients had disease-causing mutations, including 80 (63.0%) patients diagnosed by Sanger sequencing, 40 (31.5%) by TES, and 4 (3.1%) by WES.

Like most genetic disorders, hereditary SRNS shows ethnic and geographic differences. However, there have been no large-scale genetic studies for pediatric patients with SRNS/FSGS in Korea. In this study, phenotype-genotype correlations were studied in a cohort of Korean pediatric patients with SRNS/FSGS.

## 2. Materials and Methods

### 2.1. Study Participants

This study was approved by the Institutional Review Board of Seoul National University Hospital (IRB No. 0812-002-264). Informed consent was obtained from all individual participants and/or their parents. All methods were carried out in accordance with the Declaration of Helsinki. Two hundred ninety-one pediatric patients with an onset age of renal symptoms ≤20 years were recruited from 20 major pediatric nephrology centers in Korea. All patients had persistent proteinuria (spot urine protein/creatinine ratio >0.2 mg/mg) or fully developed NS. Most patients showed resistance to conventional oral steroid therapy either at the initial presentation (initial steroid non-responders) or during follow-up (late steroid non-responders). Based on the KDIGO (Kidney Disease: Improving Global Outcomes) clinical practice guideline for management of glomerulonephritis [15], an initial non-responder is defined as “failure to achieve complete remission after 8 weeks of corticosteroid therapy”, and a late non-responder is defined as “persistent proteinuria during 4 or more weeks of corticosteroids following one or more remissions”. Some patients did not receive steroid treatment because they showed congenital onset, advanced stage of chronic kidney disease (CKD), or mild to moderate degree of proteinuria only at the initial presentation. Medical records of the patients were reviewed retrospectively.

### 2.2. Strategy of Mutational Studies

In most patients, screening of a single or few candidate genes using Sanger sequencing was performed first; (1) *NPHS1*, *NPHS2*, *WT1*, and *LAMB2* for patients with congenital (disease onset within the first 3 months of life) or infantile (disease onset at age 4–12 months) NS [12,13]; (2) *INF2*, *ACTN4*, and *TRPC6* for patients with a family history of autosomal dominant (AD) inheritance; (3) *NPHS2* and *NUP107* for patients with a family history of autosomal recessive (AR) inheritance; and (4) corresponding causative gene for patients with syndrome diseases. In patients suspected of mitochondrial cytopathy, especially MELAS (mitochondrial encephalopathy, lactic acidosis, and stroke-like episodes) syndrome, heteroplasmy of the 3243A > G variation in *MT-TL1* was tested by polymerase chain reaction-restriction fragment length polymorphism (PCR-RFLP) using the ApaI restriction enzyme [16]. Then, TES or WES were performed for the remaining patients and those patients with negative results in the initial screening tests.

### 2.3. Targeted Exome Sequencing (TES) and Variant Calling

The gene panel for TES covered 57 genes known to be associated with SRNS or FSGS at that time (Supplementary Table S1). Genomic DNA was extracted from whole blood using QIAamp DNA mini kits (Qiagen, Valencia, CA, USA). The quality or quantity of DNA was checked using a Nanodrop 8000 ultraviolet–visible (UV–Vis) spectrometer (Thermo Scientific, Waltham, MA, USA) and Qubit 2.0 Fluorometer (Life Technologies, Grand Island, NY, USA). Genomic DNA was sheared by a Covaris S220 (Covaris, Woburn, MA, USA). Targeted exome capture was performed using a SureSelect XT customized kit (Agilent Technologies, Santa Clara, CA, USA). Sequencing was performed on the HiSeq 2500 platform with paired-end 100-bp reads (Illumina, San Diego, CA, USA).

Sequenced reads were aligned to the human reference genome (hg19) using the Burrows–Wheeler alignment (BWA) tool with the maximal exact match (MEM) algorithm (version 0.7.5) [17]. SAMtools (version 0.1.18) [18], Picard Tools (version 1.93, https://broadinstitute.github.io/picard/, accessed on 31 January 2020) and GATK (version 3.1-3) [19] were used for manipulating aligned sequenced data. Single nucleotide variants and InDels (insertions/deletions) were detected by UnifiedGenotyper in GATK. Various information, such as mutation nomenclature, dbSNP id, minor allele frequency of ExAC (the Exome Aggregation Consortium), and LJB23 databases, were annotated for each variant by ANNOVAR (Annotate Variation) [20]. Several filtering steps were applied to obtain candidates from germline variants; (1) variants with very low VAF (<5%), (2) variants with minor allele frequency (>1%) in the 1000GP3, and (3) variants in genes not related to the disease. Interpretation of variants followed the American College of Medical Genetics and Genomics (ACMG) guidelines, and variants classified as pathogenic or likely pathogenic were considered to be disease-causing mutations after confirmation by Sanger sequencing [21].

### 2.4. Whole-Exome Sequencing (WES) and Variant Calling

The procedures used for the preparation of genomic DNA, whole-exome capture using an Agilent V5 array, sequencing using the Illumina HiSeq 2500 platform (Illumina, San Diego, CA, USA), read alignment, variant calling, variant filtering and de novo variant calling have been described previously [22].

### 2.5. Statistical Analysis

To determine significant differences between groups with or without pathogenic variants, categorical variables were analyzed using the chi-square test or Fisher’s exact test, and continuous variables were compared using the *t*-test or Mann–Whitney U test. All values are reported as the median (interquartile range, IQR). The statistical analysis was performed using SPSS version 24.0 (SPSS, Armonk, NY, USA).

## 3. Results

### 3.1. Phenotypes

The clinical features of the patients are summarized in Table 1. A total of 291 unrelated pediatric patients (male:female = 162:129) with SRNS/FSGS were recruited for this study. The median onset age was 3.9 years (IQR 1.5–9.2 years), and the median duration of follow-up from the onset was 8.0 years (IQR 3.4–13.3 years). None of the patients were offspring of a consanguineous couple, and 48 (16.4%) patients had a family history of kidney disease.

Regarding age of onset, 35 (12.2%) patients showed congenital onset, and 25 (8.6%) patients showed infantile onset. One hundred seventy-seven (60.8%) patients presented with NS, 93 (32.0%) patients with proteinuria, and 19 (6.5%) patients with CKD or ESRD. Conventional steroid therapy was administered to 206 patients, and 191 (92.7%) patients showed steroid resistance. In 83 patients, steroid therapy was not performed because they presented with congenital onset (*n* = 32), advanced stage of CKD (*n* = 29), or a mild to moderate degree of proteinuria only (*n* = 22). Kidney biopsy was performed in 236 patients and FSGS revealed in 165 (69.9%) patients, minimal change disease in 31 (13.1%) patients, and other lesions in 40 (16.9%) patients. During follow-up, 113 (38.8%) patients had maintained a normal estimated glomerular filtration rate (eGFR), 29 (10.0%) patients progressed to CKD stages 2–4, and 144 (49.5%) patients progressed to ESRD. Kidney transplantation was performed in 100 patients, including 71 patients with FSGS, nine of whom had recurrence of SRNS/FSGS in the graft kidney.

### 3.2. Genotypes

The overall detection rate of disease-causing mutations was 43.6% (127 of 291 patients): 80 (27.5%) patients by Sanger sequencing, three (1.0%) patients with MELAS syndrome by PCR-RFLP of the *MT-TL1* gene, four (1.4%) patients by WES, and 40 (13.7%) patients by TES. Among 127 patients with disease-causing mutations, 59 (46.5%) patients had AD mutations, 58 (45.7%) patients had AR mutations, and 10 (7.9%) patients had X-linked or mitochondrial mutations.

*WT1* was the most common causative gene (23.6%, 30 patients), followed by *COQ6* (8.7%, 11 patients), *NPHS1* (8.7%, 11 patients), *NUP107* (7.1%, 9 patients), *COQ8B* (6.3%, 8 patients), *MYH9* (4.7%, 6 patients), and *INF2* (4.7%, 6 patients) (Table 2). There were 14 patients with mutations in phenocopy genes, including *COL4A3–5* and *WDR19* (Table 2). The genotypes and clinical features of patients with disease-causing mutations are summarized in Supplementary Table S2. In addition, variants of unknown significance (VUS) were detected by TES in 23 additional patients (Supplementary Table S3).

### 3.3. Genotype-Phenotype Correlations

**Figure 1:**
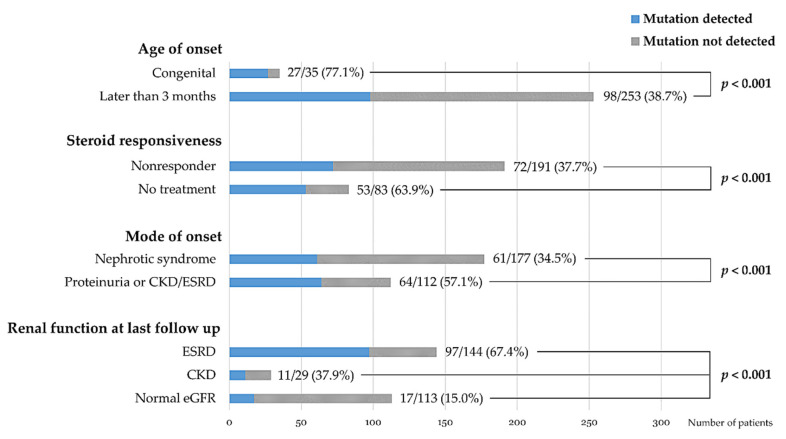
Comparison of mutation detection rates among subgroups of patients according to onset age, steroid responsiveness, mode of onset, and renal functional outcome.

A comparison between patients with disease-causing mutations and patients without mutations is summarized in Table 1. The mutation detection rate was 77.1% in patients with congenital onset, which was significantly higher than that in patients with older disease onset (38.7%, *p* < 0.001). The rate was lower in patients presenting with NS than in patients presenting with proteinuria or CKD/ESRD (34.5% versus 57.1%, *p* < 0.001). Patients who did not receive steroid treatment showed a higher mutation detection rate than steroid non-responders (63.9% versus 37.7%, *p* < 0.001). None of the steroid responders and only one of 12 steroid late non-responders had mutations. Moreover, mutations were not detected in any of the patients with minimal change disease. Interestingly, the mutation detection rate showed a negative correlation with the current eGFR of the patients: 15.0% in patients with normal eGFR, 37.9% in patients with CKD stages 2–4, and 67.4% in patients with ESRD (Table 1 and Figure 1). The rate of progression to ESRD showed no significant difference between the mutation (+) and mutation (−) groups, and disease recurrence in the graft kidney was noted in the latter group only.

**Figure 2:**
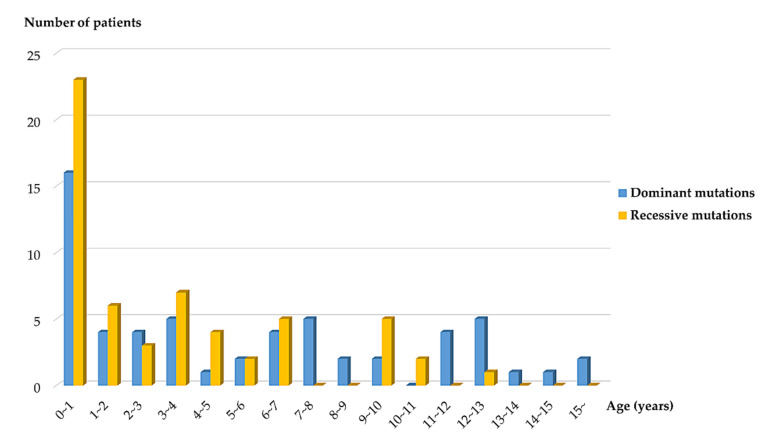
Distribution of dominant and recessive mutations by onset age.

Comparison between patients with AD mutations and patients with AR mutations showed that most (11 of 12) patients with disease onset ≥13 years had AD mutations (Supplementary Table S4 and Figure 2). Other clinical parameters showed no significant difference between the two groups.

## 4. Discussion

In this work, of 291 Korean children with SRNS or FSGS, genetic mutations were identified in 127 patients (43.6%) in one of 26 causative genes. Thirty different novel mutations detected in 15 causative genes are described in Supplemental Table S2. The mutation detection rate was higher than the 23.6–30.3% reported by previous large cohort studies from Western and Asian countries (Table 3). Such differences cannot be explained clearly, but the size of the sample database, selection bias of the target patients, and differences in ethnicity may play some role.

One of the most striking differences in this study compared with studies from other countries was the low prevalence of *NPHS2* mutations—1.4% of total patients and 3.1% of patients with mutations. In 2015, two large international cohort studies, the PodoNet Registry study [3] and the SRNS Study Group [4], using a panel of genes covering 31 and 27 candidate genes, respectively, were published. In these studies, *NPHS2* was the most commonly mutated gene (49.8% and 33.7% of patients with mutations, respectively), and the sum of three major causative genes (*NPHS2*, *NPHS1*, and *WT1*) accounted for 81.9% and 74.8% of total mutations, respectively. In 2017, another study from the United Kingdom using WES with a focus on 53 genes was published [2]. In this study, the proportion of mutations in *NPHS2* and the sum of three major causative genes (*NPHS2*, *NPHS1*, and *WT1*) were 24.5% and 61.3%, respectively. The difference between the studies published in 2015 and 2017 may be due, at least in part, to differences in the number of genes studied. An international study [23] using WES showed a low prevalence of *NPHS2* and *WT1* mutations because many enrolled individuals were screened for mutations in *WT1* and *NPHS2* before performing WES, and those who screened positive were excluded. Moreover, a Japanese study [6] showed that none of the 230 unrelated patients with proteinuria had *NPHS2* mutations, and a Chinese study [5] showed that *NPHS2* mutations were detected in 4 (3.3%) of 120 children with SRNS or isolated proteinuria, which accounted for 11.8% of 33 patients with disease-causing mutations. The lower prevalence of *NPHS2* mutations in Asian countries, including Korea, may be due to the absence of common mutations, i.e., a founder effect [4,24,25]. Another interesting finding in this study is the higher prevalence of mutations in *COQ6*, *COQ8B* and *NUP107*, which was consistent with our previous studies [26,27,28]. An increased prevalence of *COQ8B* mutations was also found in a Chinese study [5], while an increased prevalence of *NUP107* mutations was found in a Japanese study [6].

In this study, the mutation detection rate was higher in patients with congenital onset than in patients with other onset age groups (77.1% versus 38.7%, *p* < 0.001). Previous reports [4,12,13] have shown that the mutation detection rate is inversely related to age at disease onset in patients with SRNS. In a European study [12], the mutation detection rates of congenital and infantile NS were 84.8% and 44.1%, respectively (66.3% in the first year combined). Similarly, a previous study [13] from our group reported that the mutation detection rates of congenital and infantile NS were 93.9% and 20.2%, respectively (56.7% overall). In addition, a large international cohort study by the SRNS Study Group [4] reported the mutation detection rate by onset age as follows: onset in the first 3 months of life (69.4%), between 4 and 12 months old (49.7%), between 1 and 6 years old (25.3%), between 7 and 12 years old (17.8%), and between 13 and 18 years old (10.8%).

This study showed a higher mutation detection rate in patients who presented with proteinuria than in patients who presented with NS (57.0% versus 34.5%, *p* < 0.001). Similarly, in a Japanese study [6], the absence of edema was one of the risk factors for genetic mutations in pediatric patients with severe proteinuria. In this study, the mutation detection rate was higher in patients without steroid treatment than in patients with initial steroid non-responsiveness (63.9% versus 39.7%, *p* < 0.001). Most patients who did not receive steroid treatment in the present study presented with congenital onset, advanced stage CKD, or proteinuria, and these findings may contribute to the higher mutation detection rate. SRNS with AD mutations, except for *WT1* mutations, typically presents later in life, in adolescence or adulthood [4,7]. This study also showed that 10 of 11 patients with disease onset over 13 years of age had AD mutations. Disease recurrence in the graft kidney was noted in the mutation (-) group only. The recurrence rate after transplantation in the mutation (−) group (25.8%, 8 of 31 patients) was lower than that in a previous report from a Korean tertiary center (50.0%, 18 of 36 idiopathic FSGS patients) [29].

Early genetic diagnosis can benefit patients with SRNS in many ways. First, patients do not have to waste their time and money on an accurate diagnosis, i.e., a ‘diagnostic odyssey’ [14,30]. Second, genetic diagnosis is essential for family counseling. Third, detection of certain mutations allows prediction and further screening of renal and extrarenal comorbidities and helps to avoid possible future complications, e.g., risk of Wilms’ tumor or gonadoblastoma in patients with *WT1* mutations [31,32]. Fourth, patients can avoid invasive diagnostic procedures (e.g., a kidney biopsy) and ineffective, possibly harmful, drug therapy. Fifth, some rare mutations are potentially treatable with other therapeutic options, e.g., coenzyme Q10 supplementation for mutations involving the coenzyme Q10 biosynthesis pathway, such as *COQ2*, *COQ6*, *COQ8B* or *PDSS2* mutations [33,34,35]. Finally, genetic diagnosis is important for preparing a kidney transplantation in terms of donor evaluation in family members with AD mutations as well as predicting the possibility of recurrence of FSGS in the graft kidney [3,36].

Ideally, genetic testing is recommended for all children with SRNS due to the relatively high overall mutation detection rate. If it is practically unfeasible, patients at high risk of genetic mutations should be selected. Commonly applied indications for genetic testing are as follows: (1) early age of onset (congenital or infantile); (2) a family history of SRNS or consanguinity; (3) presence of typical extrarenal manifestation of syndromic diseases; (4) progression to CKD or ESRD, and (5) preoperative evaluation for kidney transplantation [7,8,9,10,11].

Fourteen (11.0% of patients with disease-causing mutations) patients in this study had mutations in four phenocopying genes, including *COL4A3–5* and *WDR19*. Because these cases manifested proteinuria or SRNS as the only evident clinical sign at disease onset or even later, they were clinically diagnosed with and treated for SRNS. A phenocopy is defined as “a phenotypic trait or disease that resembles the trait expressed by a particular genotype, but in an individual who is not a carrier of that genotype” [37]. In a recent study [23] in 300 families with SRNS using WES, 74 families (25%) had mutations in one of the known SRNS genes, and 11 families (3.7%) had mutations in genes that cause a phenocopy of SRNS. Landini et al. [38] studied 64 young patients diagnosed with SRNS, and the exome sequencing results were filtered in silico for 298 genes related to CKD, including but not limited to SRNS-related genes. They found disease-causing variants in podocytopathy genes typically associated with SRNS and FSGS in 19 patients (30%) as well as pathogenic mutations in phenocopy genes, including *COL4A3–5* (Alport syndrome), *CLCN5* (Dent disease), *PAX2* (renal-coloboma syndrome), and *GLA* (Fabry disease), in 18 patients. In addition, they found distinct extrarenal phenotypes typical of the genetic diagnoses in all patients and/or first-degree relatives by post hoc thorough ‘reverse phenotyping’. These findings highlight that, even in the era of genomics, precise and thorough phenotyping by clinicians remains essential for an accurate genetic diagnosis, including selection of the appropriate genetic testing method and interpretation of results [14,39].

## 5. Conclusions

In conclusion, the overall mutation detection rate in this cohort of Korean pediatric patients with SRNS was 43.6%. Mutations in *COQ6*, *NUP107*, and *COQ8B* were more frequently detected, and mutations in *NPHS2* were less commonly detected in this cohort compared to study cohorts from Western countries. The mutation detection rate was higher in patients with congenital onset, those who presented with proteinuria or CKD/ESRD, and those who did not receive steroid treatment. The worse the current renal function was, the higher the mutation detection rate. Genetic tests should be performed for pediatric patients with SRNS, especially for patients with younger onset age, presentation with proteinuria or progression to advanced CKD stages.

## Figures and Tables

**Table 1:** Genotype-phenotype correlations in pediatric patients with steroid-resistant nephrotic syndrome or focal segmental glomerulosclerosis.

Phenotype	Mutation (+) Patients (*n* = 127)	Mutation (−) Patients (*n* = 164)	Total Patients (*n* = 291)	Mutation Detection Rate	*p* Value
Age at onset					
Congenital	27 (21.3%)	8 (4.9%)	35 (12.0%)	77.1%	<0.001 ^b^
Infantile	11 (8.7%)	14 (8.5%)	25 (8.6%)	44.0%	
1–6 years	40 (31.5%)	74 (45.1%)	114 (39.2%)	35.1%	
7–12 years	32 (25.2%)	46 (28.0%)	78 (26.8%)	41.0%	
≥13 years	15 (11.8%)	21 (12.8%)	36 (12.4%)	41.7%	
Data unavailable	2 (1.6%)	1 (0.6%)	3 (1.0%)		
Sex ratio ^a^ (Male:female)	71:56	91:73	162:129		
Family history (+)	24 (18.9%)	24 (14.6%)	48 (16.5%)	50.0%	
Mode of onset					
Nephrotic syndrome	61 (48.0%)	116 (70.7%)	177 (60.8%)	34.5%	<0.001 ^c^
Proteinuria	53 (41.7%)	40 (24.4%)	93 (32.0%)	57.0%	
CKD/ESRD	11 (8.7%)	8 (4.9%)	19 (6.5%)	57.9%	
Data unavailable	2 (1.6%)	0	2 (0.7%)		
Steroid responsiveness					
Responder	0	15 (9.1%)	15 (5.2%)	0%	
Non-responder	72 (56.7%)	119 (72.6%)	191 (65.6%)	37.7%	
Initial non-responder	71	108	179	39.7%	
Late non-responder	1	11	12	8.3%	
No treatment	53 (41.7%)	30 (18.3%)	83 (28.5%)	63.9%	<0.001 ^d^
Data unavailable	2 (1.6%)	0	2 (0.7%)		
Renal biopsy					
FSGS	78 (61.4%)	87 (53.0%)	165 (56.7%)	47.3%	
Minimal change disease	0	31 (18.9%)	31 (10.7%)	0%	
Others	20 (15.7%)	20 (12.2%)	40 (13.7%)	50.0%	
Not done	27 (21.3%)	23 (14.0%)	50 (17.2%)	54.0%	
Data unavailable	2 (1.6%)	3 (1.8%)	5 (1.7%)		
Renal function at the last FU					<0.001 ^e^
Normal eGFR	17 (13.4%)	96 (58.5%)	113 (38.8%)	15.0%	
CKD stages 2–4	11 (8.7%)	18 (11.0%)	29 (10.0%)	37.9%	
ESRD	97 (76.4%)	47 (28.7%)	144 (49.5%)	67.4%	
Data unavailable	2 (1.6%)	3 (1.8%)	5 (1.7%)		
Duration (years) from onset to ESRD (*n* = 144)	3.6 ± 4.3	5.0 ± 4.8	4.1 ± 4.5		0.091
Recurrence after renal transplantation (*n* = 100)	0/64 (0%)	9/36 (25.0%)	9/100 (9.0%)		<0.0001

^a^ Sex of patients with *WT1* mutations and sex reversal followed by their karyotypes; ^b^ Congenital onset group (27/35, 77.1%) versus other onset age groups (98/253, 38.7%); ^c^ Nephrotic syndrome group (61/177, 34.5%) versus other mode of onset groups (64/112, 57.1%); ^d^ No steroid treatment group (53/83, 63.9%) versus steroid non-responders (72/191, 37.7%); ^e^ Among three groups: normal eGFR, CKD stage 2–4, and ESRD groups.; FSGS, focal segmental glomerulosclerosis; eGFR, estimated glomerular filtration rate; CKD, chronic kidney disease; ESRD, end-stage renal disease; FU, follow-up.

**Table 2:** Mutation screening results.

Gene	Mode of Inheritance	No. of Patients (%)	% of Total Patients (*n* = 291)
SRNS/FSGS gene			
*WT1*	AD	30 (23.6%)	10.3%
*COQ6*	AR	11 (8.7%)	3.8%
*NPHS1*	AR	11 (8.7%)	3.8%
*NUP107*	AR	9 (7.1%)	3.1%
*COQ8B*	AR	8 (6.3%)	2.7%
*MYH9*	AD	6 (4.7%)	2.1%
*INF2*	AD	6 (4.7%)	2.1%
*PAX2*	AD	5 (3.9%)	1.7%
*NPHS2*	AR	4 (3.1%)	1.4%
*MAFB*	AD	4 (3.1%)	1.4%
*LAMB2*	AR	3 (2.4%)	1.0%
*SMARCAL1*	AR	3 (2.4%)	1.0%
*MT-TL1*	Mitochondrial	3 (2.4%)	1.0%
*ACTN4*	AD	1 (0.8%)	0.3%
*LMX1B*	AD	1 (0.8%)	0.3%
*ANLN*	AD	1 (0.8%)	0.3%
*TRPC6*	AD	1 (0.8%)	0.3%
*TP53RK*	AR	1 (0.8%)	0.3%
*PODXL*	AR	1 (0.8%)	0.3%
*DGKE*	AR	1 (0.8%)	0.3%
*FOXP3*	X-linked	1 (0.8%)	0.3%
*LCAT* *COQ2*	AR AR	1 (0.8%) 1 (0.8%)	0.3% 0.3%
Subtotal		113 (89.0%)	38.8%
Phenocopying gene			
*COL4A5*	X-linked	6 (4.7%)	2.1%
*COL4A4*	AD/AR	4 (3.1%)	1.4%
*WDR19*	AR	3 (2.4%)	1.0%
*COL4A3*	AD	1 (0.8%)	0.3%
Subtotal		14 (11.0%)	4.8%
Total		127 (100%)	43.6%

AD, autosomal dominant; AR, autosomal recessive.

**Table 3:** Genetic studies in large cohorts of pediatric patients with steroid-resistant nephrotic syndrome.

	Trautmann et al., 2015 [3]	Sadowski et al., 2015 [4]	Bierzynska et al., 2017 [2]	Wang et al., 2017 [5]	Warejko et al., 2018 [23]	Nagano et al., 2020 [6]	This Study
Country	International	International	United Kingdom	China	International	Japan	Korea
Modality	GP (31 genes)	GP (27 genes)	WES (53 genes)	GP (28 genes)	WES	GP (60 genes)	Sanger/GP (57 genes) ^c^
Detection rate ^a^	277/1174 (23.6%)	526/1783 (29.5%)	49/187 (26.2%)	34/120 (28.3%)	85/300 (28.3%)	69/230 (30.0%)	127/291 (43.6%)
Commonly mutated genes ^b^	*NPHS2* 138 (49.8%)	*NPHS2* 177 (33.7%)	*NPHS1* 14 (28.6%)	*COQ8B* 8 (23.5%)	*NPHS1* 13 (15.3%)	*WT1* 17 (24.6%)	*WT1* 30 (23.6%)
	*WT1* 48 (17.3%)	*NPHS1* 131 (24.9%)	*NPHS2* 12 (24.5%)	*NPHS1* 7 (20.6%)	*PLCE1* 11 (12.9%)	*NPHS1* 8 (11.6%)	*COQ6* 11 (8.7%)
	*NPHS1* 41 (14.8%)	*WT1* 85 (16.2%)	*WT1* 4 (8.2%)	*WT1* 7 (20.6%)	*NPHS2* 8 (9.4%)	*INF2* 8 (11.6%)	*NPHS1* 11 (8.7%)
	*SMARCAL1* 12 (4.3%)	*PLCE1* 37 (7.0%)	*NUP107* 4 (8.2%)	*NPHS2* 4 (11.8%)	*SMARCAL1* 8 (9.4%)	*TRPC6* 7 (10.1%)	*NUP107* 9 (7.1%)
	*PLCE1* 10 (3.6%)	*LAMB2* 20 (3.8%)	*TRPC6* 3 (6.1%)	*LMX1B* 2 (5.9%)	*LAMB2* 6 (7.1%)	*LAMB2* 6 (8.7%)	*COQ8B* 8 (6.3%)

^a^ Overall detection rate of mutations; ^b^ The parentheses denote the percentage of total patients with mutations. ^c^ WES (*n* = 4) and polymerase chain reaction-restriction fragment length polymorphism (*n* = 3) as well; GP, gene panel; WES, whole-exome sequencing.

## Supplementary Materials

The following are available online at https://www.mdpi.com/2077-0383/9/6/2013/s1: Table S1: Fifty-seven genes included in the targeted exome sequencing, Table S2: Genotypes and phenotypes of patients with disease-causing mutations, Table S3: Variants of unknown significance detected by targeted exome sequencing, Table S4: Comparison between patients with autosomal dominant (AD) mutations and patients with autosomal recessive (AR) mutations. References [22,24,33,34,40,41,42,43,44,45,46,47,48,49,50,51,52,53,54,55,56,57,58,59,60,61,62,63,64,65,66,67,68,69,70,71,72,73,74,75,76,77,78,79,80,81,82,83,84,85,86,87,88,89,90] are cited in the Supplementary Materials.
