# Correction: Park et al. Genetic Study in Korean Pediatric Patients with Steroid-Resistant Nephrotic Syndrome or Focal Segmental Glomerulosclerosis. *J. Clin. Med.* 2020, *9*, 2013

**DOI:** 10.3390/jcm11113016

**Published:** 2022-05-27

**Authors:** Eujin Park, Chung Lee, Nayoung K. D. Kim, Yo Han Ahn, Young Seo Park, Joo Hoon Lee, Seong Heon Kim, Min Hyun Cho, Heeyeon Cho, Kee Hwan Yoo, Jae Il Shin, Hee Gyung Kang, Il-Soo Ha, Woong-Yang Park, Hae Il Cheong

**Affiliations:** 1 Department of Pediatrics, Seoul National University College of Medicine, Seoul 03080, Korea; eujinpark@hallym.or.kr (E.P.); medicalpooh@hanmail.net (Y.H.A.); kanghg@snu.ac.kr (H.G.K.); ilsooha@snu.ac.kr (I.-S.H.); 2 Department of Pediatrics, Kangnam Sacred Heart Hospital, Hallym University College of Medicine, Seoul 07441, Korea; 3 Samsung Genome Institute, Samsung Medical Center, Seoul 06351, Korea; spinelyc@gmail.com (C.L.); bionkdk@gmail.com (N.K.D.K.); woongyang.park@samsung.com (W.-Y.P.); 4 GENINUS Inc., Seoul 05836, Korea; 5 Department of Pediatrics, Asan Medical Center Children’s Hospital, University of Ulsan College of Medicine, Seoul 05505, Korea; yspark@amc.seoul.kr (Y.S.P.); pedkid@gmail.com (J.H.L.); 6 Department of Pediatrics, Pusan National University Children’s Hospital, Yangsan 50612, Korea; pedksh@gmail.com; 7 Department of Pediatrics, Kyungpook National University School of Medicine, Daegu 41944, Korea; chomh@knu.ac.kr; 8 Department of Pediatrics, Samsung Medical Center, Sungkyunkwan University School of Medicine, Seoul 06351, Korea; heeyeon1.cho@samsung.com; 9 Department of Pediatrics, Korea University Guro Hospital, Seoul 02841, Korea; guroped@korea.ac.kr; 10 Department of Pediatrics, Yonsei University College of Medicine, Seoul 03722, Korea; shinji@yuhs.ac; 11 Division of Pediatric Nephrology, Severance Children’s Hospital, Seoul 03722, Korea; 12 Department of Molecular Cell Biology, Sungkyunkwan University School of Medicine, Suwon 16419, Korea

In the original article [1], there were errors in Tables 2, 3 and S2 as published. The patient SRNS-168 was the only one having *COQ2* mutations in the study. However, it was incorrectly described as a mutation in the *COQ6* gene. The corrected Table 2, Table 3 and Table S2 appear below. Also, the sentences in Abstract and Results Section 3.2. paragraph 2 have been corrected. “*WT1* was the most common causative gene (23.6%), followed by *COQ6* (8.7%), *NPHS1* (8.7%), *NUP107* (7.1%), and *COQ8B* (6.3%).” “*WT1* was the most common causative gene (23.6%, 30 patients), followed by *COQ6* (8.7%, 11 patients), *NPHS1* (8.7%, 11 patients), *NUP107* (7.1%, 9 patients), *COQ8B* (6.3%, 8 patients), *MYH9* (4.7%, 6 patients), and *INF2* (4.7%, 6 patients) (Table 2).”

The authors apologize for any inconvenience caused and state that the scientific conclusions are unaffected. The original article has been updated.

## Figures and Tables

**Table 2:** Mutation screening results.

Gene	Mode of Inheritance	No. of Patients (%)	% of Total Patients (*n* = 291)
SRNS/FSGS gene			
*WT1*	AD	30 (23.6%)	10.3%
*COQ6*	AR	11 (8.7%)	3.8%
*NPHS1*	AR	11 (8.7%)	3.8%
*NUP107*	AR	9 (7.1%)	3.1%
*COQ8B*	AR	8 (6.3%)	2.7%
*MYH9*	AD	6 (4.7%)	2.1%
*INF2*	AD	6 (4.7%)	2.1%
*PAX2*	AD	5 (3.9%)	1.7%
*NPHS2*	AR	4 (3.1%)	1.4%
*MAFB*	AD	4 (3.1%)	1.4%
*LAMB2*	AR	3 (2.4%)	1.0%
*SMARCAL1*	AR	3 (2.4%)	1.0%
*MT-TL1*	Mitochondrial	3 (2.4%)	1.0%
*ACTN4*	AD	1 (0.8%)	0.3%
*LMX1B*	AD	1 (0.8%)	0.3%
*ANLN*	AD	1 (0.8%)	0.3%
*TRPC6*	AD	1 (0.8%)	0.3%
*TP53RK*	AR	1 (0.8%)	0.3%
*PODXL*	AR	1 (0.8%)	0.3%
*DGKE*	AR	1 (0.8%)	0.3%
*FOXP3*	X-linked	1 (0.8%)	0.3%
*LCAT* *COQ2*	AR AR	1 (0.8%) 1 (0.8%)	0.3% 0.3%
Subtotal		113 (89.0%)	38.8%
Phenocopying gene			
*COL4A5*	X-linked	6 (4.7%)	2.1%
*COL4A4*	AD/AR	4 (3.1%)	1.4%
*WDR19*	AR	3 (2.4%)	1.0%
*COL4A3*	AD	1 (0.8%)	0.3%
Subtotal		14 (11.0%)	4.8%
Total		127 (100%)	43.6%

AD, autosomal dominant; AR, autosomal recessive.

**Table 3:** Genetic studies in large cohorts of pediatric patients with steroid-resistant nephrotic syndrome.

	Trautmann et al., 2015 [3]	Sadowski et al., 2015 [4]	Bierzynska et al., 2017 [2]	Wang et al., 2017 [5]	Warejko et al., 2018 [23]	Nagano et al., 2020 [6]	This Study
Country	International	International	United Kingdom	China	International	Japan	Korea
Modality	GP (31 genes)	GP (27 genes)	WES (53 genes)	GP (28 genes)	WES	GP (60 genes)	Sanger/GP (57 genes) ^c^
Detection rate ^a^	277/1174 (23.6%)	526/1783 (29.5%)	49/187 (26.2%)	34/120 (28.3%)	85/300 (28.3%)	69/230 (30.0%)	127/291 (43.6%)
Commonly mutated genes ^b^	*NPHS2* 138 (49.8%)	*NPHS2* 177 (33.7%)	*NPHS1* 14 (28.6%)	*COQ8B* 8 (23.5%)	*NPHS1* 13 (15.3%)	*WT1* 17 (24.6%)	*WT1* 30 (23.6%)
	*WT1* 48 (17.3%)	*NPHS1* 131 (24.9%)	*NPHS2* 12 (24.5%)	*NPHS1* 7 (20.6%)	*PLCE1* 11 (12.9%)	*NPHS1* 8 (11.6%)	*COQ6* 11 (8.7%)
	*NPHS1* 41 (14.8%)	*WT1* 85 (16.2%)	*WT1* 4 (8.2%)	*WT1* 7 (20.6%)	*NPHS2* 8 (9.4%)	*INF2* 8 (11.6%)	*NPHS1* 11 (8.7%)
	*SMARCAL1* 12 (4.3%)	*PLCE1* 37 (7.0%)	*NUP107* 4 (8.2%)	*NPHS2* 4 (11.8%)	*SMARCAL1* 8 (9.4%)	*TRPC6* 7 (10.1%)	*NUP107* 9 (7.1%)
	*PLCE1* 10 (3.6%)	*LAMB2* 20 (3.8%)	*TRPC6* 3 (6.1%)	*LMX1B* 2 (5.9%)	*LAMB2* 6 (7.1%)	*LAMB2* 6 (8.7%)	*COQ8B* 8 (6.3%)

^a^ Overall detection rate of mutations; ^b^ The parentheses denote the percentage of total patients with mutations. ^c^ WES (*n* = 4) and polymerase chain reaction-restriction fragment length polymorphism (*n* = 3) as well; GP, gene panel; WES, whole-exome sequencing.

**Table S2:** Genotypes and phenotypes of patients with disease-causing mutations.

Gene	Patient ID	Mutations	Age at Onset (Years)	Sex ^b^	Family History	Mode of Onset	Kidney Biopsy	Renal Outcome	Time to ESRD (Years)
*WT1* (REFSEQ: NM_024426.5)									
	SRNS-20	c.1400G > A, p.R467Q	At birth	M	N	NS	ND	ESRD	0.1
	SRNS-42	c.1136delT, p.V379Dfs ^a^	6.5	M	N	PU	FSGS	ESRD	2.7
	SRNS-126	c.1231C > T, p.H411Y ^a^	1.5	F	N	NS	FSGS	ESRD	7.3
	SRNS-151	c.1315C > T, p.R439C	At birth	M	N	NS	ND	ESRD	0.0
	SRNS-156	c.760C > A, p.P254T ^a^	3.3	M	N	NS	FSGS	ESRD	6.1
	SRNS-186	c.1363C > T, p.P455S	8.0	M	Y	NS	FSGS	Normal eGFR	NA
	SRNS-222	c.1316G > A, p.R439H	At birth	M	N	NS	FSGS	ESRD	0.4
	SRNS-223	c.1316G > A, p.R439H	At birth	F	N	NS	DMS	ESRD	0.0
	SRNS-224	c.1316G > A, p.R439H	At birth	F	N	NS	FSGS	Death	NA
	SRNS-225	c.1316G > A, p.R439H	At birth	F	N	NS	ND	ESRD	0.0
	SRNS-226	c.1316G > C, p.R439P	At birth	M	NA	PU	ND	ESRD	1.8
	SRNS-227	c.1315C > T, p.R439C	At birth	F	N	NS	ND	ESRD	0.0
	SRNS-228	c.1324C > A, p.Q442K	1.0	F	N	ESRD	ND	ESRD	0.0
	SRNS-229	c.1372T > A, p.C458S	At birth	M	N	NS	DMS	ESRD	1.1
	SRNS-230	c.1399C > T, p.R467W	At birth	M	N	PU	DMS	ESRD	1.8
	SRNS-231	c.1405G > T, p.D469Y	At birth	F	NA	NS	DMS	ESRD	0.0
	SRNS-232	c.785 − 1G > C in intron 2 ^a^	NA	M	NA	NA	ND	NA	NA
	SRNS-233	c.1447 + 4C > T in intron 9	6.6	F	N	NS	FSGS	CKD	NA
	SRNS-234	c.1447 + 4C > T in intron 9	2.6	F	N	NS	FSGS	Normal eGFR	NA
	SRNS-235	c.1447 + 4C > T in intron 9	3.5	M	N	ESRD	ND	ESRD	0.0
	SRNS-236	c.1447 + 4C > T in intron 9	6.8	M	N	NS	MesPGN	ESRD	15.4
	SRNS-237	c.1447 + 4C > T in intron 9	At birth	F	N	CKD	ND	ESRD	0.7
	SRNS-238	c.1447 + 5G > A in intron 9	At birth	M	N	PU	FSGS	ESRD	19.7
	SRNS-239	c.1447 + 5G > A in intron 9	5.0	F	N	NS	FSGS	ESRD	4.2
	SRNS-240	c.1447 + 5G > A in intron 9	6.8	F	N	PU	FSGS	ESRD	12.9
	SRNS-241	c.1447 + 5G > A in intron 9	11.4	M	N	CKD	FSGS	ESRD	0.3
	SRNS-242	c.1447 + 5G > A in intron 9	12.3	M	N	PU	FSGS	Normal eGFR	NA
	SRNS-243	c.1419_1430del12, p.H474_T477del ^a^	At birth	M	N	ESRD	ND	ESRD	0.0
	SRNS-244	c.1381T > C, p.C461R	2.2	F	N	ESRD	MesPGN	ESRD	0.0
	SRNS-245	c.1297T > C, p.C433R	At birth	F	N	ESRD	ND	ESRD	0.0
*COQ6* (REFSEQ: NM_182476.2)									
	SRNS-61	c.686A > C, p.Q229P c.782C > T, p.P261L	1.1	M	N	PU	FSGS	Normal eGFR	NA
	SRNS-103	c.124G > T, p.G42C ^a^ c. 782C > T, p.P261L	At birth	F	N	NS	FSGS	ESRD	0.4
	SRNS-203	c.484C > T, p.R162* c.782C > T, p.P261L	9.1	M	N	PU	FSGS	ESRD	0.8
	SRNS-251	c.189_191del3, p.K64del c.782C > T, p.P261L	3.9	M	N	NS	FSGS	ESRD	2.2
	SRNS-252	c.189_191del3, p.K64del c.686A > C, p.Q229P	2.0	F	N	NS	FSGS	ESRD	1.1
	SRNS-253	c.189_191del3, p.K64del c.782C > T, p.P261L	3.9	F	N	NS	FSGS	ESRD	0.1
	SRNS-254	c.189_191del3, p.K64del c.782C > T, p.P261L	2.7	F	N	NS	FSGS	ESRD	1.9
	SRNS-255	c.189_191del3, p.K64del c.782C > T, p.P261L	1.2	F	Y	NS	FSGS	ESRD	0.1
	SRNS-256	c.189_191del3, p.K64del c.782C > T, p.P261L	3.1	M	N	NS	FSGS	ESRD	0.4
	SRNS-257	c.686A > C, p.Q229P c.782C > T, p.P261L	At birth	M	N	NS	FSGS	ESRD	1.7
	SRNS-258	c.189_191del3, p.K64del c.782C > T, p.P261L	1.1	M	N	NS	FSGS	ESRD	0.2
*NPHS1* (REFSEQ: NM_004646.3)									
	SRNS-85	c.2156_2163del8, p.L719Pfs*4 c.2464G > A, p.V822M	At birth	F	Y	NS	MesPGN	Death	0.0
	SRNS-206	c.2156_2163del8, p.L719Pfs*4 c.3250dupG, p.V1084Gfs*12	At birth	M	N	NS	MesPGN	ESRD	2.5
	SRNS-207	c.2442C > G, p.Y814* c.1379G > A, p.R460Q	At birth	F	N	NS	ND	ESRD	3.0
	SRNS-208	c.188A > G, p.Q63R c.1885G > T, p.E629*	At birth	M	N	NS	FSGS	ESRD	1.6
	SRNS-209	c.3027C > G, p.Y1009* c.3478C > T, p.R1160*	At birth	F	N	NS	ND	ESRD	3.2
	SRNS-210	c.2765C > A, p.A922D c.3287 − 11G > A in intron 24	At birth	M	N	NS	FSGS	CKD	NA
	SRNS-211	c.2156_2163del8, p.L719Pfs*4 c.3478C > T, p.R1160*	At birth	M	N	NS	ND	ESRD	4.7
	SRNS-212	c.58 + 2T > C in intron 1 ^a^ c.1338delT, p.I466Mfs*16 ^a^	At birth	F	Y	NS	MesPGN	ESRD	1.5
	SRNS-213	c.526 + 1G > A in intron 4 c.1632_1634del3, p.545del	At birth	M	N	NS	ND	Normal eGFR	NA
	SRNS-214	c.3213dupG, p.L1072Afs*24 ^a^ c.3478C > T, p.R1160*	At birth	M	N	NS	ND	Death	0.0
	SRNS-215	c.139delG, p.A47Pfs*81 (homozygote)	At birth	M	N	NS	MesPGN	ESRD	1.8
*NUP107* (REFSEQ: NM_020401.3)									
	SRNS-71	c.934delT, p.Y312Tfs ^a^ c.2492A > C, p.D831A	4.8	M	N	PU	FSGS	ESRD	8.7
	SRNS-259	c.2071C > T, p.Q691* c.2492A > C, p.D831A	4.3	M	Y	NS	FSGS	ESRD	4.2
	SRNS-260	c.627_663dup37, p.L225Ffs*15 ^a^ c.2492A > C, p.D831A	3.8	F	N	PU	FSGS	ESRD	3.0
	SRNS-261	c.1079_1083del5, p.E360Gfs*6 c.2492A > C, p.D831A	3.4	M	Y	NS	FSGS	ESRD	2.0
	SRNS-262	c.1079_1083de5l, p.E360Gfs*6 c.2492A > C, p.D831A	2.4	M	N	PU	FSGS	ESRD	2.7
	SRNS-263	c.1079_1083del5, p.E360Gfs*6 c.2492A > C, p.D831A	3.8	M	N	ESRD	ND	ESRD	0.0
	SRNS-264	c.469G > T, p.D157Y c.2492A > C, p.D831A	10.9	F	Y	CKD	FSGS	ESRD	2.1
	SRNS-265	c.1079_1083del5, p.E360Gfs*6 c.2492A > C, p.D831A	4.0	F	N	ESRD	ND	ESRD	0.0
	SRNS-266	c.2492A > C, p.D831A c.1735 − 3T > G in intron 20	4.1	M	Y	PU	FSGS	ESRD	7.4
*COQ8B* (REFSEQ: NM_024876.3)									
	SRNS-25	c.759C > A, p.N253K (homozygote)	1.1	F	NA	NS	FSGS	ESRD	1.5
	SRNS-35	c.737G > A, p.S246N c.532C > T, p.R178W	6.7	M	N	PU	FSGS	CKD	NA
	SRNS-93	c.737G > A, p.S246N c.1548C > A, p.Y516* ^a^	9.9	F	N	PU	FSGS	Normal eGFR	NA
	SRNS-246	c.449G > A, p.R150Q c.759C > A, p.N253K	5.1	M	Y	PU	FSGS	ESRD	5.1
	SRNS-247	c.737G > A, p.S246N c.759C > A, p.N253K	10.8	F	N	PU	FSGS	ESRD	2.0
	SRNS-248	c.737G > A, p.S246N (homozygote)	9.2	F	N	PU	FSGS	ESRD	3.0
	SRNS-249	c.737G > A, p.S246N c.1468C > T, p.R490C	6.9	F	N	PU	FSGS	ESRD	3.9
	SRNS-250	c.737G > A, p.S246N (homozygote)	13.0	F	N	PU	FSGS	Normal eGFR	NA
*MYH9* (REFSEQ: NM_002473.5)									
	SRNS-205	c.3494G > T, p.R1165L	16.8	F	Y	PU	ND	ESRD	17.5
	SRNS-273	c. 2152C > T, p.R718W	1.3	M	N	NS	MesPGN	ESRD	5.3
	SRNS-274	c.287C > T, p.S96L	20.0	M	N	PU	FSGS	ESRD	0.7
	SRNS-275	c.287C > T, p.S96L	12.1	F	N	PU	ND	ESRD	8.3
	SRNS-276	c.2104C > T, p.R702C	8.7	F	NA	PU	MesPGN	ESRD	7.8
	SRNS-277	c.287C > T, p.S96L	12.4	M	NA	PU	MesPGN	ESRD	10.5
*INF2* (REFSEQ: NM_022489.3)									
	SRNS-63	c.233T > G, p.L78R ^a^	11.0	M	N	PU	FSGS	ESRD	6.7
	SRNS-69	c.658G > A, p.E220K	11.1	F	N	PU	FSGS	ESRD	4.0
	SRNS-268	c.658G > A, p.E220K	7.4	M	Y	PU	FSGS	ESRD	5.8
	SRNS-269	c.658G > A, p.E220K	11.7	M	N	NS	FSGS	ESRD	5.5
	SRNS-270	c.230T > C, p.L77P	9.2	F	N	NS	FSGS	ESRD	3.4
	SRNS-271	c.529C > T, p.R177C	12.6	F	Y	PU	FSGS	Normal eGFR	NA
*PAX2* (REFSEQ: NM_003987.4)									
	SRNS-26	c.76dupG, p.V26Gfs*28	5.3	M	N	PU	FSGS	ESRD	10.2
	SRNS-31	c.563A > G, p.N188S ^a^	3.4	M	N	NS	ND	Normal eGFR	NA
	SRNS-32	c.222_225dup4, p.G76Dfs ^a^	13.4	M	Y	PU	FSGS	CKD	NA
	SRNS-95	c.74G > A, p.G25E ^a^	7.2	F	N	PU	FSGS	ESRD	7.3
	SRNS-191	c.419G > A, p.R140Q	7.8	M	N	PU	FSGS	Normal eGFR	NA
*NPHS2* (REFSEQ: NM_014625.3)									
	SRNS-27	c.503G > A, p.R168H c.467dupT, p.L156Ffs*11	1.3	F	NA	NS	ND	ESRD	3.6
	SRNS-47	c.412C > T, p.R138* c.503G > A, p.R168H	2.1	M	N	NS	FSGS	ESRD	4.9
	SRNS-136	c.502C > T, p.R168C c.851C > T, p.A284V	6.9	M	N	PU	FSGS	CKD	NA
	SRNS-216	c.358T > C, p.S120P c.503G > A, p.R168H	At birth	M	N	NS	FSGS	ESRD	8.4
*COL4A5* (REFSEQ: NM_000495.4)									
	SRNS-49	c.834 + 1G > A in intron 14	10.0	F	Y	PU	FSGS	CKD	NA
	SRNS-81	c.956G > A, p.G319D	10.1	M	Y	PU	FSGS	ESRD	10.1
	SRNS-87	c.4946delT, p.L1649Rfs*4 ^a^	12.9	M	Y	PU	FSGS	ESRD	6.7
	SRNS-120	c.1165 + 1G > A in intron 19	3.8	M	N	NS	FSGS	ESRD	6.7
	SRNS-134	c.4082T > A, p.L1361* ^a^	14.0	M	Y	PU	FSGS	ESRD	8.4
	SRNs-190	c.4532G > A, p.R1511H	12.8	M	N	PU	FSGS	CKD	NA
*COL4A4* (REFSEQ: NM_000092.4)									
	SRNS-53	c.1111delG, p.D371Tfs ^a^ c.1323_1340del18, p.P444_L449del	0.8	F	Y	PU	MesPGN	ESRD	18.9
	SRNS-148	c.2630G > A, p.R877Q	3.6	M	N	NS	ND	Death	2.3
	SRNS-152	c.1046G > A, p.R349Q ^a^	2.5	F	N	NS	FSGS	Normal eGFR	NA
	SRNS-181	c.2630G > A, p.R877Q	14.3	F	N	PU	FSGS	Normal eGFR	NA
*MAFB* (REFSEQ: NM_005461.4)									
	SRNS-204	c.194G > T, p.S65I	9.8	M	Y	PU	ND	Normal eGFR	NA
	SRNS-280	c.183C > A, p.S61R	12.5	F	N	PU	FSGS	CKD	NA
	SRNS-281	c.211C > G, p.P71A	4.4	M	N	PU	FSGS	ESRD	0.6
	SRNS-282	c.212C > T, p.P71L	1.2	M	N	PU	ND	Normal eGFR	NA
*LAMB2* (REFSEQ: NM_002292.3)									
	SRNS-217	c.1503_1504delAT, p.C502* c.4267delT, p.C1423Vfs*29	0.7	F	N	NS	FSGS	ESRD	10.8
	SRNS-218	c.2283-2286del4, p.S762Rfs*29 c.536C > T, p.S179F	At birth	F	N	NS	FSGS	CKD	NA
	SRNS-219	c.474delT, p.A159Pfs*33 ^a^ c.1328_1329del2, p.H443Rfs*11 ^a^	At birth	F	N	NS	ND	ESRD	0.1
*WDR19* (REFSEQ: NM_025132.3)									
	SRNS-289	c.3533G > A, p.R1178Q c.3703G > A, p.E1235K	9.6	M	N	PU	FSGS	ESRD	1.4
	SRNS-290	c.3533G > A, p.R1178Q c.3703G > A, p.E1235K	6.2	F	Y	PU	MesPGN	ESRD	3.0
	SRNS-291	c.1853T > C, p.L618P c.3533G > A, p.R1178Q	At birth	M	N	CKD	ND	ESRD	0.3
*SMARCAL1* (REFSEQ: NM_014140.3)									
	SRNS-144	c.1682G > A, p.R561H c.1851 + 1G > T in intron 9 ^a^	6.0	M	N	NS	FSGS	ESRD	3.4
	SRNS-287	c.1411dupA, p.I471Nfs ^a^ c.1484A > C, p.Q495P ^a^	5.5	F	N	NS	FSGS	ESRD	1.5
	SRNS-288	c.1484A > C, p.Q495P ^a^ c.1851 + 1G > T in intron 9 ^a^	3.5	M	N	PU	FSGS	ESRD	2.1
*MT-TL1* (REFSEQ: NC_012920)									
	SRNS-284	mtDNA3243A > G	18.9	F	Y	PU	DMS	CKD	NA
	SRNS-285	mtDNA3243A > G	11.8	F	Y	PU	FSGS	ESRD	6.0
	SRNS-286	mtDNA3243A > G	9.8	F	N	PU	FSGS	ESRD	5.3
*FOXP3* (REFSEQ: NM_014009.3)									
	SRNS-283	c.736 − 2A > G in intron 7 ^a^	3.4	M	N	NS	MNP	Normal eGFR	NA
*ACTN4* (REFSEQ: NM_004924.5)									
	SRNS-267	c.785C > T, p.S262F	3.5	M	Y	NS	FSGS	ESRD	1.2
*LMX1B* (REFSEQ: NM_002316.3)									
	SRNS-279	c. 668G > A, p.R223Q	2.1	F	N	NS	FSGS	ESRD	1.86
*ANLN* (REFSEQ: NM_018685.4)									
	SRNS-65	c.2305A > T, p.L769* ^a^	7.7	M	N	PU	FSGS	Normal eGFR	NA
*TRPC6* (REFSEQ: NM_004621.5)									
	SRNS-37	c.523C > G, p.R175G ^a^	8.5	F	N	PU	FSGS	ESRD	2.3
*COL4A3* (REFSEQ: NM_000091.4)									
	SRNS-199	c.4793T > G, p.L1598R	0.5	F	N	NS	DMS	ESRD	0.9
*TP53RK* (REFSEQ: NM_033550.3)									
	SRNS-221	c.194A > T, p.K65M (homozygote)	At birth	F	NA	NA	ND	Death	0.0
*DGKE* (REFSEQ: NM_003647.2)									
	SRNS-272	c.501C > G, p.C167W c.610dupA, p.T204Nfs*4	0.5	M	N	PU	FSGS	CKD	NA
*LCAT* (REFSEQ: NM_000229.1)									
	SRNS-278	c.794_801del8, p.E265Afs*18 c.931delT, p.F311Lfs*99 ^a^	9.6	M	Y	PU	FSGS	Normal eGFR	NA
*COQ2* (REFSEQ: NM_015697.7)									
	SRNS-168	c.392A > G, p.D131G ^a^ c.518G > A, p.R173H ^a^	At birth	F	N	NS	FSGS	ESRD	0.3
*PODXL* (REFSEQ: NM_005397.3)									
	SRNS-220	c.3G > T, p.M1? c.926G > A, p.W309*	At birth	M	Y	NS	ND	ESRD	0.0

^a^ Novel mutations. ^b^ Sex of patients with WT1 mutations and sex reversal, followed by their karyotypes. NA, not available; ND, not done; NS, nephrotic syndrome; PU, proteinuria; CKD, chronic kidney disease; ESRD, end-stage renal disease; eGFR, estimated glomerular filtration rate; FSGS, focal segmental glomerulosclerosis; DMS, diffuse mesangial sclerosis; MesPGN, mesangial proliferative glomerulonephritis; MNP, membranous nephropathy; M, male; F, female; Y, yes; N, no.
